# Distribution characteristics of fungi on surfaces in the hospital environment: What have we overlooked?

**DOI:** 10.1371/journal.pone.0360522

**Published:** 2026-11-04

**Authors:** Wenbin He, Fan Cheng, Xiaoyan Chen, Nuo Chen, Ying Zhang, Yan Li, Yibin Tan, Wenwen Wu, Bilong Feng, Xiaofeng Lin, Xiaokang Huang, Weilong Zhou, Ying Wang

**Affiliations:** 1 Department of Colorectal and Anal Surgery, Zhongnan Hospital of Wuhan University, Wuhan, China; 2 Department of Infection Prevention and Control Management, Zhongnan Hospital of Wuhan University, Wuhan, China; 3 Department of Nursing, Zhongnan Hospital of Wuhan University, Wuhan, China; 4 School of Public Health, Hubei University of Medicine, Shiyan, China; 5 Health Science Center, Yangtze University, Jingzhou, China; 6 Sinopharm Dongfeng General Hospital, Hubei University of Medicine, Shiyan, China; 7 Hubei Engineering Center for Infectious Disease Prevention, Control and Treatment, Wuhan, China; 8 Department of Logistics Support, Zhongnan Hospital of Wuhan University, Wuhan, China; 9 Department of Infection Prevention and Control, West China Women's and Children’s Hospital, Chengdu, China; City University of New York, UNITED STATES OF AMERICA

## Abstract

Hospital environmental fungal contamination is a significant cause of healthcare-associated fungal infections, which are associated with high mortality rates in immunocompromised populations. However, current understanding of the distribution characteristics and high-risk colonization sites of fungi on hospital inanimate surfaces remains limited, hindering the development of targeted infection control strategies. A cross-sectional study was conducted in December 2024 in a large tertiary care hospital in central China. Using two-stage stratified sampling, 100 environmental surface samples were collected from 10 clinical departments. All samples underwent fungal culture and quantification, and culture-positive samples were further analyzed by 18S rRNA sequencing to characterize fungal community composition. A total of 20% of samples were culture-positive for fungi, and 7% exceeded the ECDC-recommended threshold of 5 colony-forming units per square centimeter (CFU/cm²). Water-related surfaces showed the highest culture-positive rate (32.1%), with the highest individual count observed in an endocrinology department handwashing pool (15 CFU/cm^2^), followed by air disinfection machine vents and faucets. *Aspergillus* was the dominant genus (43.8% of isolates), and 70% of identified genera included WHO-listed priority pathogenic fungi. Water-related surfaces and ventilation equipment outlets are high-risk sites for fungal colonization. Current cleaning protocols are insufficient for these areas, requiring enhanced targeted surveillance and disinfection to reduce healthcare-associated fungal infection risk.

## 1. Introduction

Healthcare-associated infections (HAIs) are one of the most significant and unavoidable public health problems today. The ubiquitous nature of pathogenic microorganisms in hospital environments contributes significantly to HAIs. Various studies have indicated that the transmission of pathogenic microorganisms in hospital environments accounts for approximately 10–20% of HAI cases [1]. While bacterial HAIs have been extensively studied, fungal healthcare-associated infections have emerged as a rapidly growing threat in recent decades [2].

Globally, an estimated 6.55 million individuals are at risk of fungal diseases annually, with over 3.75 million fatalities recorded; among these deaths, mycoses directly account for 2.55 million cases [3]. The economic burden of fungal infections surpasses $7.2 billion in the United States [4]. Although fungal HAIs are reported less frequently than bacterial HAIs, their high mortality rates and limited treatment options pose a substantial global public health challenge owing to the constant emergence of drug-resistant strains. In 2022, the World Health Organization (WHO) identified and categorized 19 priority fungal pathogens into three groups. Cryptococcus neoformans, Candida auris, *Aspergillus* fumigatus, and Candida albicans are classified under the critical group [5], emphasizing the urgent need for global efforts in preventing and controlling fungal infections in medical institutions.

Fungi are ubiquitous in the environment and can be transmitted through multiple routes, including air, water, contact with contaminated surfaces, and human-to-human transmission [6]. Hospital environments provide favorable conditions for fungal colonization, including constant temperature and humidity control, frequent moisture exposure in water-related areas, and frequent surface contact by healthcare workers and patients. These conditions, combined with the high prevalence of immunocompromised patients in tertiary hospitals, create a high-risk setting for fungal transmission [7]. Previous studies have confirmed that hospital air and surface fungal contamination is associated with an increased risk of invasive fungal infections in high-risk patient groups, particularly in transplantation, hematology, and intensive care units [8,9].

However, current research on hospital fungal contamination has several critical gaps. Most existing studies focus on air fungal contamination, while the distribution characteristics of fungal colonization on inanimate environmental surfaces remain poorly characterized [10,11]. Previous surface contamination studies have primarily focused on bacterial pathogens, with limited data on fungal community composition and high-risk colonization sites [12,13]. Disinfection efficacy against fungal biofilms is less studied compared to bacterial biofilms. Recent research indicates that oxidizing disinfectants such as hydrogen peroxide and sodium hypochlorite have demonstrated superior efficacy in eradicating bacterial biofilms compared to membrane-active quaternary ammonium compounds [14], but the effectiveness of these disinfectants against fungal biofilms on hospital surfaces remains unclear.

The above knowledge gaps significantly impede the accurate assessment of fungal exposure risk in healthcare settings and the implementation of targeted prevention and control measures for high-risk patient populations. Therefore, this study aimed to systematically investigate the distribution characteristics, dominant fungal taxa, and high-risk colonization sites of fungal contamination on environmental surfaces across different clinical departments in a large tertiary care hospital in central China. The findings of this study will provide evidence-based guidance for optimizing hospital environmental cleaning and disinfection protocols, identifying high-priority sites for enhanced monitoring, and ultimately reducing the risk of healthcare-associated fungal infections.

## 2. Materials and methods

### 2.1. Study design

This cross-sectional investigation was conducted from 7th to 23rd December 2024 within a major tertiary care hospital with 3,300 beds. A purposive two-stage sampling method was employed to identify environmental marker points for inclusion in the study. In the first sampling stage, we intentionally selected wards susceptible to fungal infections, such as immunocompromised patient wards, based on our previous systematic review of fungal distribution in hospital environments. Finally, 10 departments susceptible to fungal infections were included as research sites (listed in Section 2.2.1). In the second sampling stage, 100 specimens were collected from 19 environmental object categories from the monitoring sites. For an in-depth analysis, the sample specimens were classified into three groups: water-related (group 1), environment-related (group 2), and sanitary ware-related (group 3). The locations of these points are shown in Fig 1.

**Fig 1:**
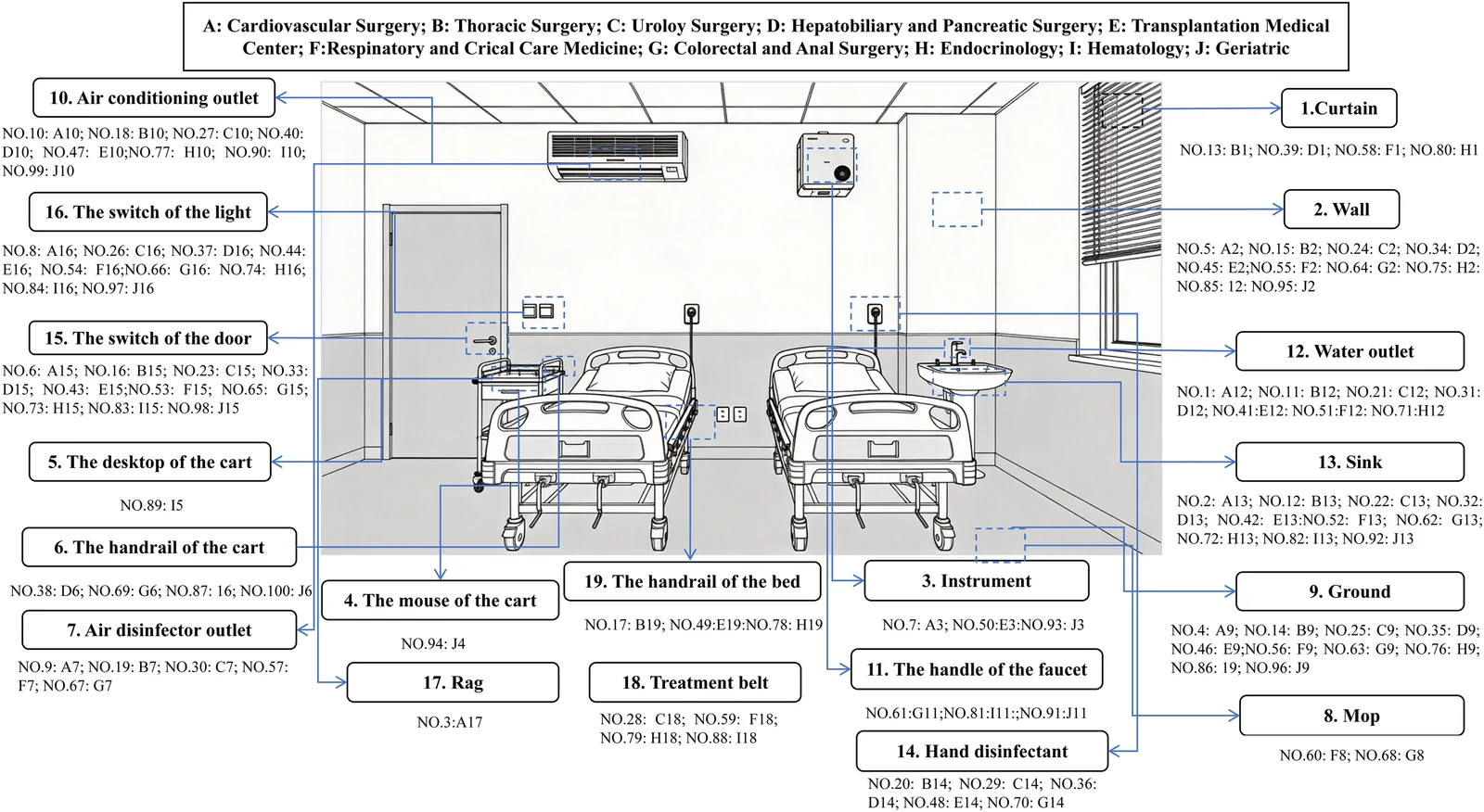
Distribution of sampling points. (Distribution of sampling points across 10 departments (A–J) and 19 surface categories (1–19) in the hospital).

### 2.2. Sample collection

#### 2.2.1. Sampling design.

This study employed a purposive two-stage sampling strategy to investigate fungal contamination on hospital environmental surfaces.

Stage 1-Selection of departments: Ten clinical departments were purposively selected based on: clinical significance (departments with immunocompromised patients), diversity of patient populations, practical accessibility for sampling, and representation of different medical specialties (surgical, medical, and specialized units). The selected departments were: cardiovascular surgery, thoracic surgery, urology surgery, hepatobiliary and pancreatic surgery, transplantation medical center, respiratory and critical care medicine, colorectal and anal surgery, endocrinology, hematology, and geriatrics. Stage 2-Selection of sampling sites: Within each department, sampling sites were selected based on: high-frequency contact surfaces, moisture-prone areas (water-related surfaces), previously reported high-contamination sites in the literature, and practical accessibility during routine hospital operations.

#### 2.2.2. Sampling scope and limitations.

A total of 100 environmental samples were collected from the 10 departments. Ten samples were collected per department (10 departments × 10 samples = 100), with sampling sites selected to represent different surface categories within each department. The sampling was not designed to be comprehensive or representative of all possible surfaces within each department.

#### 2.2.3. Sample collection procedure.

Environmental surfaces were sampled using cotton swabs following the Chinese national standard WS/T 512–2016 [15]. A 5 cm × 5 cm sterilised template was placed on the target surface, and a swab soaked with sterile 0.03 mol/L phosphate-buffered saline or normal saline was applied five times with rotation. For surfaces with residual disinfectant, the sampling solution was supplemented with neutralizers (1% sodium thiosulfate for chlorine-based disinfectants; 3% Tween 80 + 0.3% lecithin for quaternary ammonium compounds) per WS/T 512–2016. For small objects (e.g., doorknobs), swabs were applied directly. Sampling was conducted during working hours (9:00–17:00) in winter (7–23 December 2024) to minimise post-disinfection effects. All samplers received uniform training on standard operating procedures to ensure consistency. All samples were transported to the laboratory within 2 h of collection. The complete workflow from sample collection to sequencing is illustrated in S1 Fig.

### 2.3. Sample detection and analysis

Upon collection, the cotton swabs were placed in a test tube containing 10 mL eluent. The tubes were sealed in sterile bags and transported to the laboratory within 2 h. Upon arrival at the laboratory, samples were immediately centrifuged at 12,000 RPM for 10 min at 4°C, and 200 μL of the pellet was transferred to a new 1.5 mL Eppendorf tube. After vortex mixing, 100 μL of the suspension was evenly spread onto Sabouraud dextrose agar (SDA) plates supplemented with chloramphenicol (0.05 g/L) to inhibit bacterial growth. The plates were incubated at 28°C for 2–7 days, and colony counting was performed daily until day 7. Colony counts were expressed as CFU/cm² based on the sampled surface area. For culture-positive samples, fungal colonies were collected for DNA extraction and sequencing as described in Section 2.4. This study followed the principles set forth in the Declaration of Helsinki and was approved by the Clinical Research Ethics Committee of Zhongnan Hospital of Wuhan University (approval number: 2023136K).

### 2.4. DNA extraction and sequencing

Genomic DNA was extracted from swab samples and fungal colonies using the DNeasy PowerSoil Kit (Qiagen, Germany) following the manufacturer’s protocol. Briefly, samples were transferred to PowerBead Tubes containing lysis buffer and homogenized using a vortex adapter for 10 min. After centrifugation, the supernatant was mixed with binding buffer and loaded onto spin columns. The columns were washed twice with wash buffer, and purified DNA was eluted in 50 μL of elution buffer. DNA concentration and purity were assessed using a NanoDrop ND-1000 spectrophotometer (Thermo Fisher Scientific, USA). For the 16 selected samples, DNA yields ranged from 1.5 to 45.2 ng/μL (A260/A280: 1.8–2.0), corresponding to total DNA of 75–2260 ng per sample. The V4 hypervariable region of the 18S rRNA gene was amplified using primers 528F (5’-GCGGTAATTCCAGCTCCAA-3’) and 706R (5’-AATCCRAGAATTTCACCTCT-3’). PCR was performed in 25 μL reactions containing 12.5 μL 2 × Master Mix, 0.5 μL each primer (10 μM), and 1 μL template DNA (10–50 ng), with thermal cycling: 94°C for 3 min; 35 cycles of 94°C for 30 s, 55°C for 30 s, 72°C for 45 s; and 72°C for 5 min. PCR products were verified on 2% agarose gel and purified using the GeneJET Gel Extraction Kit (Thermo Fisher Scientific). Sequencing libraries were prepared using the NEBNext Ultra II DNA Library Prep Kit (NEB, USA) with 10–50 ng of purified PCR product as input, and paired-end sequenced (2 × 300 bp) on an Illumina MiSeq platform.

Of the 100 collected samples, 22 yielded visible colonies on SDA plates (culture-positive rate: 22%). Among these, 16 samples with colony counts ≥ 1 CFU/cm² were selected for 18S rRNA gene sequencing, as samples below this threshold yielded insufficient DNA (concentration < 1 ng/μL after extraction) for reliable library construction. These 16 samples covered all three surface categories (water-related, n = 9; sanitation-related, n = 3; ventilation-related, n = 4) and all 10 sampled departments. The remaining 6 culture-positive samples with colony counts < 1 CFU/cm² were excluded from sequencing due to insufficient biomass. No human participants were involved in this study. The 18S rRNA gene sequencing was performed on DNA extracted from individual fungal colonies obtained from culture-positive samples, rather than directly from environmental swabs. Therefore, the sequencing results reflect the taxonomic identity of culturable fungi and may not capture the full fungal diversity present in the original environmental samples.

### 2.5. Data analysis

Descriptive data are presented as mean ± standard deviation. All statistical analyses were performed using SPSS 26.0 (IBM Corp., Armonk, NY, USA), with visualizations generated using GraphPad Prism 8.0.1 and R v4.3.1 (ggplot2 package). For culture-based data: Contamination positivity rates across the three surface categories were compared using Pearson’s Chi-square test, with Fisher’s exact test used when expected cell counts were <5. Pairwise comparisons were adjusted using Bonferroni correction. Colony count distributions were compared using the non-parametric Kruskal-Wallis H test, with post-hoc Mann-Whitney U tests for pairwise comparisons. Odds ratios (OR) and 95% confidence intervals (CI) were calculated to quantify effect sizes. Significance was defined as a two-tailed P < 0.05. For amplicon sequencing data: Raw reads were processed using a standard bioinformatics pipeline: quality filtering (Trimmomatic v0.39), paired-end merging (FLASH v1.2.11), chimera removal (UCHIME v4.2.40), and operational taxonomic unit (OTU) clustering at 97% sequence similarity (UPARSE v7.0). Taxonomic classification was performed using the RDP classifier (confidence threshold 0.7) against the UNITE v9.0 and SILVA v138.1 databases, with taxa <0.5% relative abundance excluded from downstream analysis. Alpha and beta diversity analyses were conducted using the R vegan package v2.5.3.

## 3. Results

### 3.1. Number of colonies in the samples

Among the 100 sampled surfaces across 10 hospital departments, 20 samples (20%) were culture-positive, of which 7 (7%) exceeded the ECDC threshold of 5 CFU/cm^2^ [16] for high-risk surface cleanliness, while 13 samples had colony counts below this threshold (ranging from 1 to 4 CFU/cm^2^). Sixteen samples with colony counts ≥ 1 CFU/cm^2^ were further subjected to 18S rRNA gene sequencing. The colony counts and sequencing results for all 20 culture-positive samples are presented in Table 1. A total of 28 fungal isolates were obtained from the 20 culture-positive samples by subculturing individual colonies on SDA plates. These isolates were subsequently identified by 18S rRNA gene sequencing of the V4 region, revealing the presence of 10 distinct fungal genera across the 16 successfully sequenced samples (12 samples yielded single isolates, and 4 samples yielded multiple morphologically distinct isolates). It is important to note that this proportion applies only to the specific surfaces sampled and does not represent the prevalence of contamination across all hospital surfaces. All 10 sampled departments had at least one sampling site with colony counts exceeding the threshold. Notably, among the sampled sites, the handwashing pool in the endocrinology department exhibited the highest colony count (15 CFU/cm^2^), followed by the air disinfection machine exit in the thoracic surgery department (10 CFU/cm^2^), and the faucet in the endocrinology department (10 CFU/cm^2^). Descriptive comparison across surface categories showed that water-related surfaces had a higher descriptive positivity rate (32.1%) compared to sanitary ware-related surfaces (15.2%) and environment-related surfaces (18.2%). Similarly, median colony counts were higher for water-related surfaces (3 CFU/cm^2^) compared to sanitary ware-related surfaces (1 CFU/cm^2^) and environment-related surfaces (2 CFU/cm^2^). These descriptive patterns suggest a trend of higher fungal contamination on moisture-prone surfaces.

**Table 1:** Colony counts and 18S rRNA sequencing results of 20 culture-positive environmental surface samples.

Specimen Number	Department	Monitoring sites	CFU/cm^2^
A12	Cardiovascular surgery	water outlet	5
A17	Cardiovascular surgery	rag	3
B12	Thoracic surgery	water outlet	6
B2	Thoracic surgery	wall	4
B7	Thoracic surgery	air disinfector outlet	10
C10	Urology surgery	air conditioning outlet	3
D2	Hepatobiliary and pancreatic surgery	wall	3
D10	Hepatobiliary and pancreatic surgery	air conditioning outlet	2
E13	Transplantation medical center	sink	2
F7	Respiratory and critical care medicine	air disinfector outlet	3
F8	Respiratory and critical care medicine	mop	5
G11	Colorectal and anal surgery	the handle of the faucet	2
G9	Colorectal and anal surgery	ground	4
G8	Colorectal and anal surgery	mop	7
H12	Endocrinology	water outlet	10
H13	Endocrinology	sink	15
I11	Hematology	the handle of the faucet	4
I9	Hematology	ground	9
J11	Geriatrics	the handle of the faucet	5
J13	Geriatrics	sink	4

Note: Samples with colony count ≥ 1 CFU/cm² (n = 16) were subjected to 18S rRNA sequencing; samples with < 1 CFU/cm² were not sequenced due to insufficient DNA yield.

The overall fungal positive rate across all 100 samples was 20.0% (20/100). Statistical analysis revealed significant differences in positive rates across the three surface categories (χ^2^ = 6.72, df = 2, P = 0.035) (Table 2). Water-related surfaces had the highest positive rate (32.1%, 9/28), followed by ventilation equipment outlets (28.6%, 10/35), while sanitary ware-related surfaces had the lowest positive rate (10.8%, 4/37). Post-hoc pairwise comparisons with Bonferroni correction showed that both water-related surfaces (P = 0.027) and ventilation equipment outlets (P = 0.042) had significantly higher positive rates than sanitary ware-related surfaces, with no significant difference between the two high-risk categories (P = 0.764). Similarly, fungal colony counts varied significantly across surface types (Kruskal-Wallis H = 12.36, df = 2, P = 0.002). Water-related surfaces had the highest average colony count (6.8 ± 3.2 CFU/cm^2^), followed by ventilation equipment outlets (5.7 ± 2.9 CFU/cm^2^), both significantly higher than that of sanitary ware-related surfaces (2.1 ± 1.5 CFU/cm^2^) (P < 0.05 for both comparisons).

**Table 2:** Fungal contamination characteristics across different environmental surface types.

Surface category	Number of samples	Number of positive samples	Positive rate (%)	Average colony count (CFU/cm²)
Water-related surfaces	28	9	32.1^*^	6.8 ± 3.2^*^
Ventilation equipment outlets	35	10	28.6^*^	5.7 ± 2.9^*^
Sanitary ware-related surfaces	37	4	10.8	2.1 ± 1.5

Note: *P < 0.05 compared with sanitary ware-related surfaces (Bonferroni-corrected post-hoc test). Overall comparisons across surface categories were statistically significant for positive rate (χ² = 6.72, df = 2, P = 0.035) and colony count (Kruskal-Wallis H = 12.36, df = 2, P = 0.002).

### 3.2. Overview of the sequencing data

Sixteen culture-positive samples were subjected to 18S rRNA sequencing. A summary of fungal contamination status by department is provided in S1 Table. All 10 departments had detectable fungal contamination, with positive rates ranging from 10% to 20% across departments. As these samples were selected based on positive culture results, the following taxonomic findings apply only to the cultivable fungal community in culture-positive samples, and do not represent the total fungal diversity in the hospital environment.

Among the cultured fungal isolates from these positive samples, *Aspergillus* was the most frequently occurring fungal genus detected in 7 samples (43.8% of sequenced samples), which includes WHO critical priority pathogens (A. fumigatus, A. niger) causing invasive aspergillosis in immunocompromised patients. *Meyerozyma*, *Kodamaea* (including emerging catheter-related pathogen K. ohmeri) and Saccharomyces were each detected in 4 samples (25.0%), all with known opportunistic pathogenic potential in susceptible hosts. Less frequent genera include *Candida* (WHO critical priority pathogen), *Penicillium*, *Cladosporium*, and *Alternaria*, all associated with allergic reactions or opportunistic infections (Fig 2).

**Fig 2:**
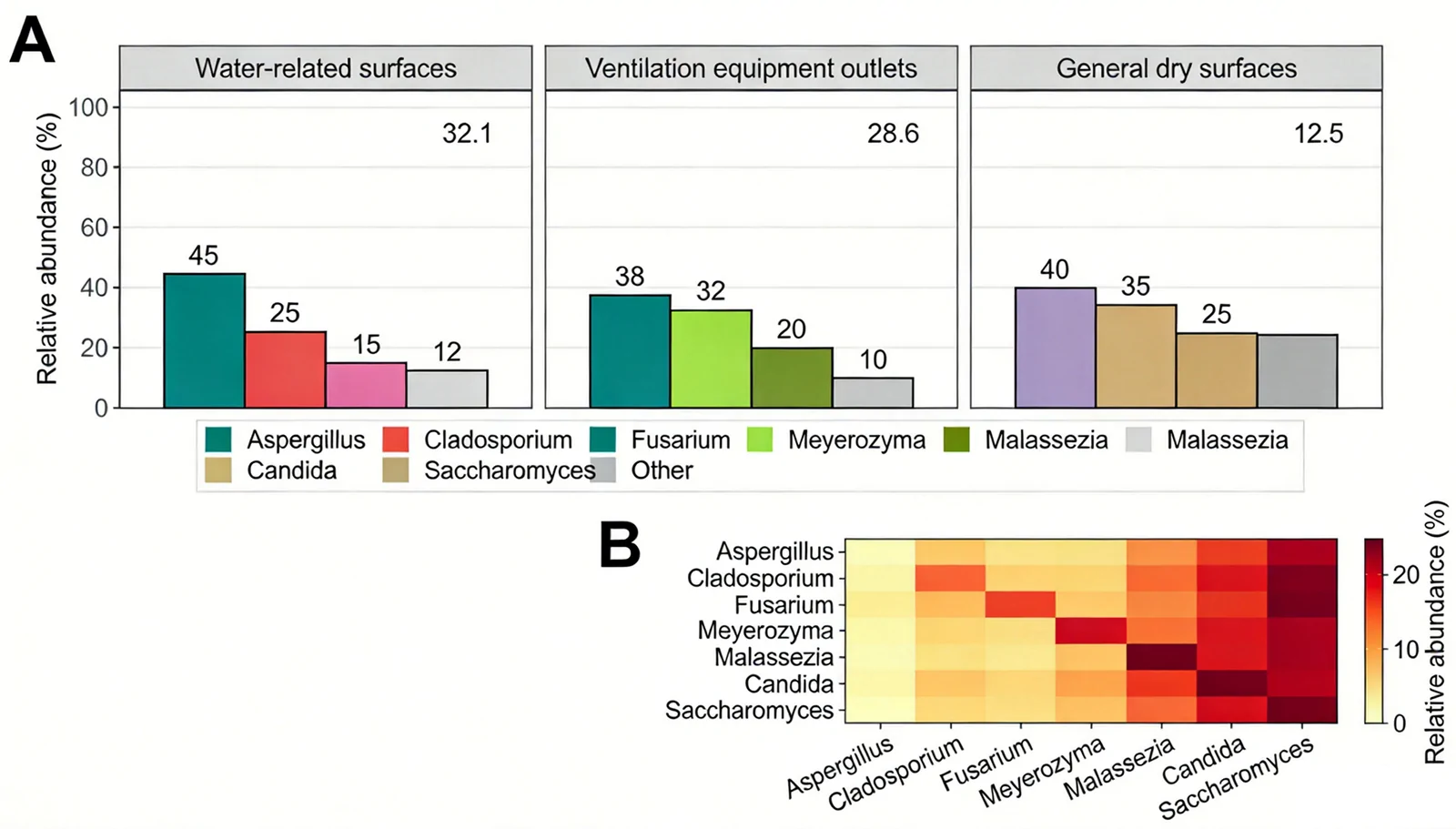
Fungal community composition at the genus level in culture-positive samples. (A) Relative abundance of the top 10 fungal genera across three surface categories (water-related surfaces, ventilation equipment outlets, and sanitary ware-related surfaces). (B) Heatmap showing the distribution of dominant fungal genera across individual samples.

### 3.3. Alpha diversity

Alpha diversity was evaluated using four primary metrics: Shannon’s diversity index, Simpson’s diversity index, evenness, and the Chao1 richness estimator. Among these samples, the Shannon diversity index ranged from 0 to 1.75, with significantly higher diversity observed in sink surface samples compared to other surface types (p < 0.05). These diversity results are representative only of culture-positive samples with detectable fungal growth. As shown in Table 1 and Fig 3, four samples (A12, B12, H12, and D10) exhibited zero values for evenness, Shannon, Chao1, and Simpson indices because of the presence of only one taxon per sample. Conversely, H13 and B7 demonstrated relatively high values for Shannon, Simpson, and evenness indices, whereas the remaining samples displayed comparatively lower levels of alpha diversity.

**Fig 3:**
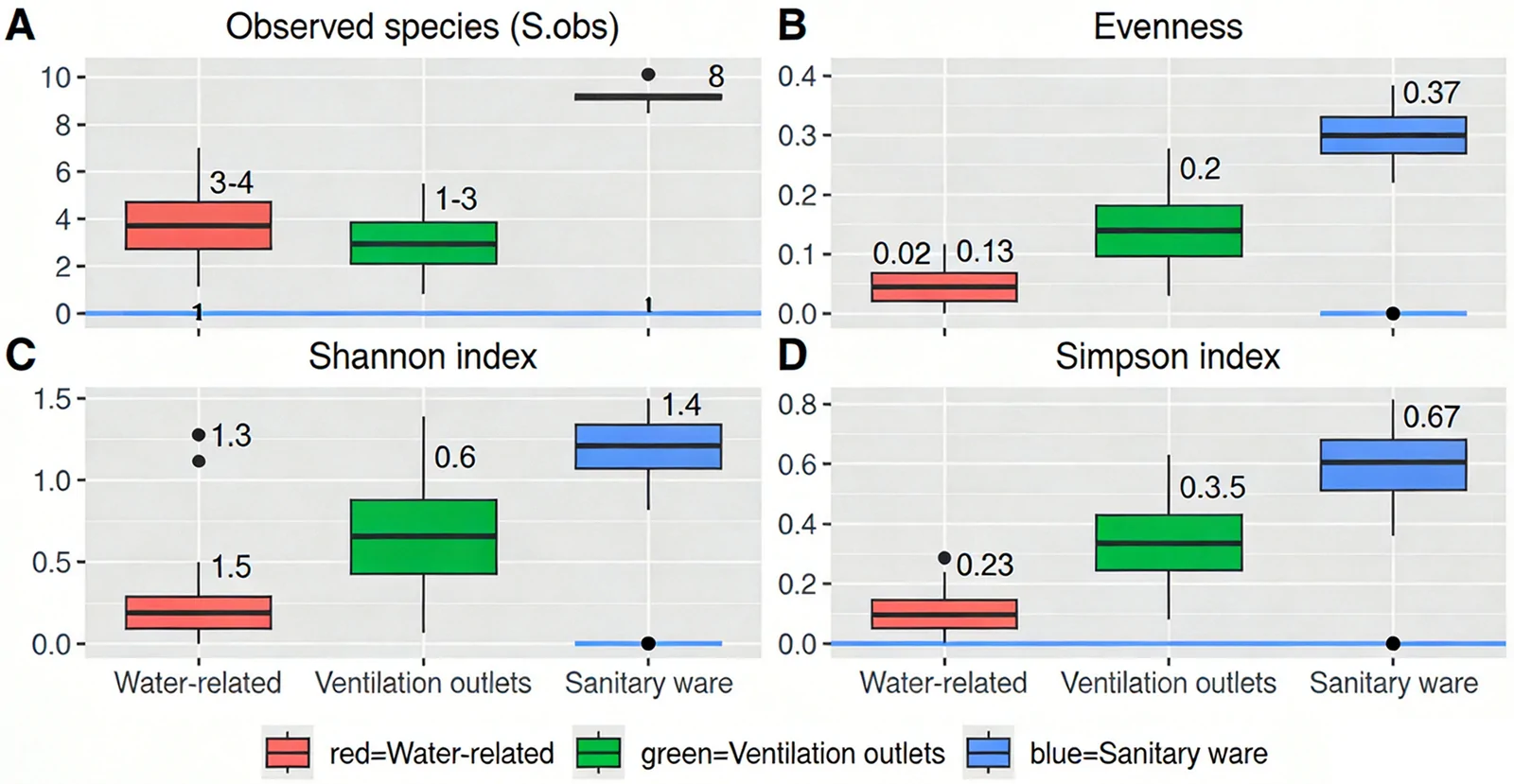
Alpha diversity. Note: The alpha diversity of the fungal communities was detected among three sampling environments: Groups 1, 2, and 3 represented water-related surfaces, ventilation equipment outlets, and sanitary ware-related surfaces, respectively.

Given the limited number of samples across different departments and locations, further comparisons of alpha diversity were conducted among samples from distinct environments. Owing to the solitary sample in group 3, a Wilcoxon rank test was selected over the Kruskal–Wallis test, which assumes a minimum of five samples per group. The alpha diversity, as indicated by the Shannon and Simpson indices, did not exhibit significant variation between groups 1 and 2 (P-values: 0.513 and 0.624, respectively).

### 3.4. Beta diversity

As shown in Fig 4, non-metric multidimensional scaling based on the Bray–Curtis dissimilarity was employed to visualise the differences in fungal communities across departments, locations, and environments (Fig 4). Due to the limited number of samples in the department and location groups, an analysis of similarities was conducted. The results indicated no significant differences between groups 1 and 2 (P-value = 0.572).

**Fig 4:**
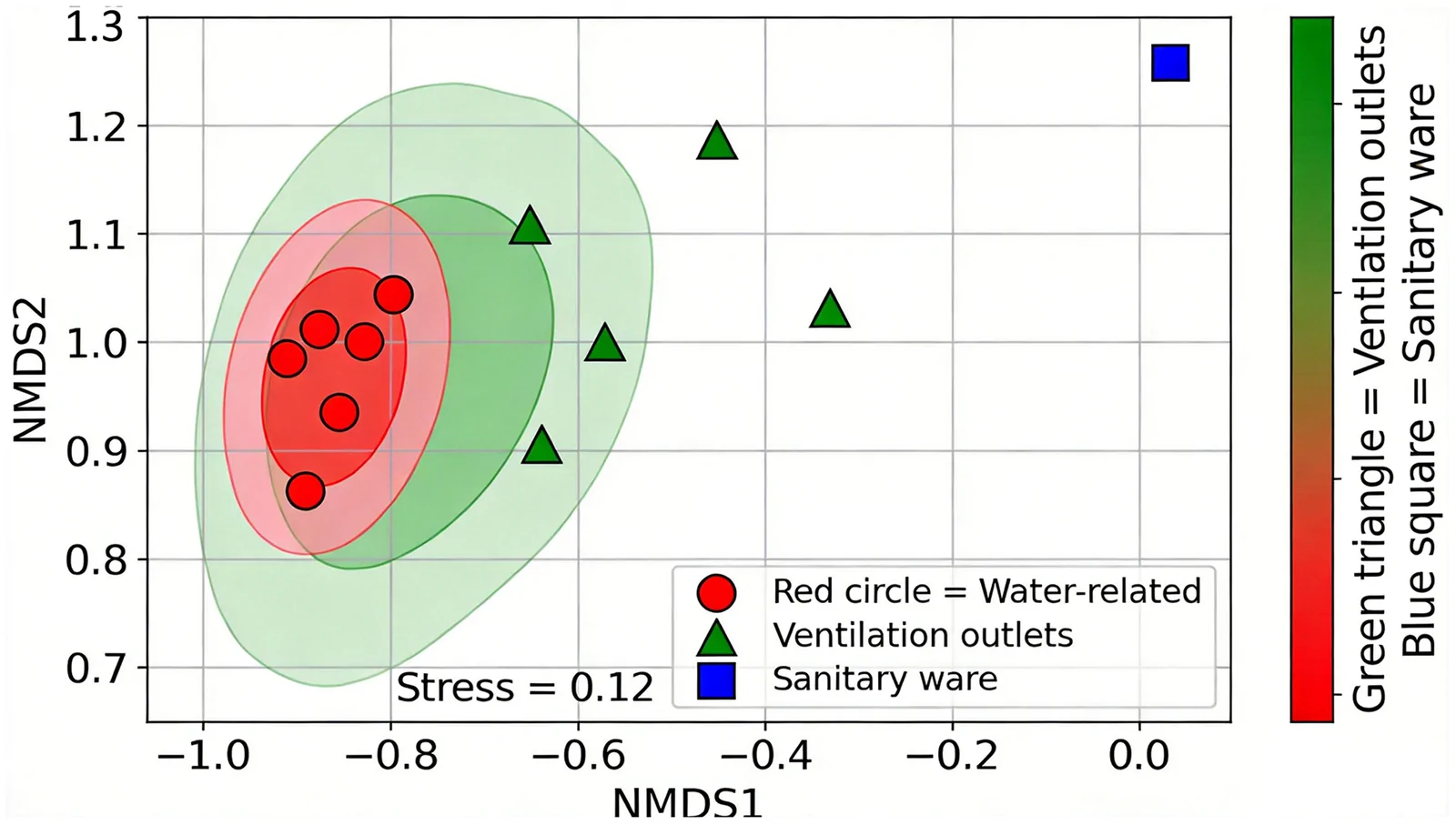
Beta diversity. Note: Non-metric multidimensional scaling (NMDS) of Bray–Curtis dissimilarity for fungal communities presented among three sampling environments: Groups 1, 2, and 3 represented water-related surfaces, ventilation equipment outlets, and sanitary ware-related surfaces.

## 4. Discussion

This study investigated the distribution characteristics of fungal contamination on environmental surfaces across 10 clinical departments in a large tertiary care hospital in central China during winter. The findings provide important insights into high-risk sites for fungal colonization and have direct implications for hospital infection control practices.

### 4.1. Key findings of fungal contamination in the hospital environment

The overall fungal contamination rate of 20% (for samples with >5 CFU/cm^2^) observed in this study is within the range of 5.8–28.6% reported in recent international hospital surface monitoring studies [17,18]. All 10 sampled departments had detectable fungal contamination, with positive rates ranging from 10% to 20%, indicating widespread fungal presence across different clinical settings. The highest contamination levels were found on water-related surfaces, with a 32.1% positivity rate and median colony count of 3 CFU/cm^2^, consistent with previous reports showing moisture-prone areas as hotspots for fungal growth [10,19]. Consistent with our hypothesis, statistical analyses revealed a significant overall difference in contamination rates and colony counts across surface categories (p < 0.05). Water-related surfaces had higher contamination rates and higher fungal loads compared to other surface types, supporting the conclusion that moisture-prone surfaces are more prone to fungal colonization in hospital environments. While some pairwise comparisons did not reach statistical significance after adjustment for multiple testing, the overall trend and effect size estimates provide support for the practical importance of these differences.

*Aspergillus* was the most frequently detected genus (43.8% of sequenced samples), which aligns with global hospital environmental surveillance data where *Aspergillus* accounts for 30–50% of all fungal isolates [20]. Importantly, 70% of the identified fungal genera include known opportunistic pathogenic species consistent with WHO priority pathogen lists, including *Aspergillus fumigatus*, *A. niger*, and *Candidaspecies*. These pathogens are a leading cause of invasive fungal infections in immunocompromised patients, with mortality rates exceeding 40% for invasive aspergillosis [20], highlighting the clinical relevance of our findings.

### 4.2. Mechanisms underlying fungal colonization on different surface types

The higher susceptibility of water-related surfaces to fungal colonization can be explained by two key factors. First, over 80% of the fungal genera identified in this study (including *Aspergillus*, *Penicillium*, *Cladosporium*, and yeast genera) are hydrophilic or xerotolerant species that require moisture for spore germination and mycelial growth. These fungi can utilize trace water remaining on sink surfaces, faucet aerators, and plumbing fixtures as a growth substrate even after routine cleaning. Second, water-related surfaces are frequently exposed to water splashes and often remain damp for extended periods between cleaning cycles, providing optimal humidity conditions for fungal proliferation. The organic matter (soap residues, skin cells, etc.) that accumulates on these surfaces further provides nutrients to support fungal growth [21,22]. Additionally, the biofilms commonly formed on plumbing outlets create a protected microenvironment that shields fungi from disinfection agents, facilitating persistent colonizationation [23].

The detection of fungal contamination on air disinfection machine vents and air conditioning outlets is also noteworthy. These sites are often not included in routine surface cleaning protocols, allowing accumulation of dust and fungal spores over time. The centralized HVAC system in the hospital, which maintains standardized temperature (22 ± 2°C) and relative humidity (40–50%) across all wards, may facilitate the dissemination of fungal spores from these ventilation-associated sites to other areas of the hospital [24], explaining the relatively homogeneous fungal community composition across departments observed in the beta diversity analysis.

### 4.3. Public health implications for hospital infection control

The findings of this study have several important implications for hospital infection control practices. First, the detection of high-priority fungal pathogens on environmental surfaces, particularly in high-risk departments with immunocompromised patient populations (transplantation center, hematology ward, respiratory ICU) [25], highlights the need for enhanced targeted monitoring of fungal contamination in these settings. The presence of *Aspergillus* species, which can cause life-threatening infections in susceptible patients, warrants particular attention [25]. Second, the identification of water-related surfaces and ventilation equipment outlets as high-risk sites for fungal colonization suggests that current standard cleaning protocols may be less effective for moisture-exposed surfaces and ventilation systems, highlighting the need for enhanced targeted surveillance and disinfection strategies for these high-risk areas [24]. We recommend that hospitals: (1) increase the frequency of cleaning and disinfection for sinks, faucets, and other water-related surfaces, with special attention to thorough drying after cleaning; (2) include air disinfection machine vents, air conditioning outlets, and other ventilation-associated surfaces in regular cleaning schedules; (3) implement periodic fungal monitoring programs for high-risk wards to identify contamination events early. Third, the mixed source profile of fungal contamination, with 85% of identified genera being common outdoor air mycobiota components and 15% being human/plumbing-associated species, indicates that a multifaceted intervention strategy is required. This should include both improved ventilation system filtration to reduce outdoor air intrusion and enhanced surface cleaning protocols to eliminate indoor fungal reservoirs.

### 4.4. Study limitations and future research directions

This study has several limitations that should be considered when interpreting the results. First, the study was conducted only during the winter season, and fungal contamination levels may vary across seasons due to changes in temperature, humidity, and ventilation patterns. Second, the sample size was relatively small, with only 1–3 positive samples per department, which limited the ability to conduct formal statistical comparisons across departments and surface types. Third, sequencing was limited to culture-positive samples only and was performed at the genus level, which prevented strain-level tracking of fungal transmission between different hospital areas. Finally, negative controls were not included during sample collection and processing, which should be considered when interpreting low-abundance taxa results.

Future research should address these limitations by: (1) conducting multi-season sampling to assess temporal variations in fungal contamination; (2) incorporating site-specific microenvironmental parameter measurements to better understand factors influencing fungal growth; (3) using species-level and strain-level sequencing approaches to characterize fungal transmission networks within the hospital; and (4) implementing appropriate negative controls at all stages of sample collection and processing to improve result accuracy. Despite these limitations, this study provides important baseline data on fungal contamination in Chinese hospital environments and identifies high-risk sites that require enhanced infection control attention.

## 5. Conclusions

Fungi-related HAIs are a global focal point of concern, with a heightened risk of infection outbreaks due to the use of communal medical devices and shared environments within healthcare settings. Our diversity analysis of culture-positive samples indicates that water-related surfaces harbor diverse fungal communities, including several opportunistic pathogens. As only culture-positive samples were analyzed, further studies including all samples regardless of culture status are needed to characterize the full environmental fungal diversity in these settings.

## Supporting information

S1 Fig Sample collection and processing workflow. (PNG)

S1 Table Summary of fungal isolation results by department. (DOCX)

S2 Table The relative abundance of each detected bacterial species at each location. (CSV)

S3 Table The sum of the relative abundances at each point position. (CSV)

S4 Table The diversity of colonies at each point position. (CSV)
