# Supplementary material for: Distribution characteristics of fungi on surfaces in the hospital environment: What have we overlooked?
Source: PLoS One. 2026 Nov 4;21(11):e0360522. doi: 10.1371/journal.pone.0360522 (PMC13734468; doi:10.1371/journal.pone.0360522)
Supplement: Supplementary file 1 [file pone.0360522.s002.docx]

**Table S1. Summary of fungal isolation results by department.**

| **Department** | **Number of samples collected** | **Positive rate (%)** | **Median CFU/cm² (IQR)** | **Isolated fungal genera** |
| --- | --- | --- | --- | --- |
| Cardiovascular surgery | 10 | 10.0 | 5.0 (3.0-5.0) | Aspergillus, Meyerozyma |
| Thoracic surgery | 10 | 20.0 | 6.0 (4.0-10.0) | Aspergillus, Penicillium, Cladosporium |
| Urology surgery | 10 | 10.0 | 3.0 (3.0-3.0) | Alternaria |
| Hepatobiliary and pancreatic surgery | 10 | 10.0 | 3.0 (2.0-3.0) | Saccharomyces |
| Transplantation medical center | 10 | 10.0 | 2.0 (2.0-2.0) | Kodamaea |
| Respiratory and critical care medicine | 10 | 20.0 | 4.0 (3.0-5.0) | Aspergillus, Penicillium |
| Colorectal and anal surgery | 10 | 20.0 | 4.0 (2.0-7.0) | Candida, Saccharomyces |
| Endocrinology | 10 | 20.0 | 12.5 (10.0-15.0) | Aspergillus, Meyerozyma, Kodamaea |
| Hematology | 10 | 20.0 | 6.5 (4.0-9.0) | Aspergillus, Cladosporium |
| Geriatrics | 10 | 20.0 | 4.5 (4.0-5.0) | Saccharomyces, Candida |
